# Hydrops Uteri as a Sequel to Imperforate Vagina

**Published:** 1890-09

**Authors:** William G. Martin

**Affiliations:** Kankakee, Ill.


### HYDROPS UTERI, AS A SEQUEL TO IMPERFORATE VAGINA.

By William G. Martin, V.S.,
Kankakee, Ill.

In the Spring of 1888 I was called to see a bay mare unable to foal. On examination I found the head between the forelegs, with the chin resting on the sternum. After rectifying the position of the foal, which was dead, delivery was effected very easily. The usual precautions as to after-treatment were taken, and the mare made a good recovery, becoming through the Summer very fleshy. At the beginning of Winter the owner called and informed me that he thought his mare was wormy, because of late she had not been doing well, and was, as he termed it, "pot-bellied." A prescription for some worm powders was given him. In two weeks he made his appearance at my infirmary with the mare, now very much emaciated, with the abdomen enormously distended. On examination she showed the following symptoms : Pulse slow and weak ; visible mucous membrane of a pale yellow tinge ; temperature, 101¼° F. Defecation was performed with much difficulty, only one or two hard balls being passed at a time ; her appetite was poor. At first I suspected abdominal pregnancy, but upon the owner assuring me that the mare had not been bred this was given up. I now decided to make an examination per vaginam. On passing my hand into the vagina, I was surprised to find it completely occluded behind the sphincter vesicæ of the bladder. It now became manifest that I had a case of imperforate vagina, and the distension of the abdomen was due to the retained fluid in the womb. Having decided to operate, the mare was secured in the standing position ; a large cattle trocar and canula were introduced into the vagina and the sac punctured. On withdrawing the trocar, it was followed by a profuse discharge of a creamy fluid, like milk. The canula being removed, I cut through the vaginal adhesions with a probe-pointed embryotomy knife, making a good bell opening to the os uteri. This was followed by a great discharge of fluid—in all, about twenty gallons were removed. The womb was now syringed out with a hot solution of bichloride of mercury, 1 to 1000, by means of a long rubber tube introduced into the uterus. The removal of the fluid was followed by severe shock, which was combatted with diffusible stimulants, and the body was enveloped in hot blankets. She rallied very slowly. The next day the vagina was packed with antiseptic cotton wool, in order to prevent adhesion again of the vaginal walls. The mare did well after the first week, when the owner, to avoid further expense, took her home. I cautioned him in regard to the closing of the vaginal walls, but the advice was disregarded. The mare gained rapidly in flesh, and was, to all appearance, restored to perfect health, as during the following Summer she worked every day on the farm. All precautions to keep the vagina open were disregarded, with the result that in October, 1889, the mare was returned to the infirmary in the same condition as the previous year, only there was more fluid present. A doubtful prognosis was now given, but the owner insisted on having the fluid removed, which was done—the mare dying five hours afterward, of acute metro-peritonitis—the amount of fluid contained in the womb being not far from twenty-five gallons. On post-mortem examination, the vagina was found to be even more completely closed by fibrous adhesions than the previous year. The womb had the appearance of being foiled and was almost divested of its lining membrane, of which great flakes were found floating in the contained fluid.

During an extensive practice of twelve years this is the first case of imperforate vagina I have met with. Dr. H. C. Allinson (in the *British Medical Journal*) reports a case somewhat analogous to mine, where an imperforate hymen resulted in the production of abdominal swelling the size of a gravid uterus at six months. The patient, a 17-year old girl, had never menstruated, but urinary retention recurred every month. The vaginal orifice was closed by the hymen, which was tense and very thin. The dark purplish fluid looked as if it would soon break through. Incision of the hymen was followed by peritonitis, from which the patient finally recovered.

